# High spatial frequencies disrupt conscious visual recognition: evidence from an attentional blink paradigm

**DOI:** 10.1016/j.heliyon.2022.e11964

**Published:** 2022-11-29

**Authors:** Martial Mermillod, Mickaël J.R. Perrier, Adeline Lacroix, Louise Kauffmann, Carole Peyrin, Alain Méot, Nicolas Vermeulen, Frédéric Dutheil

**Affiliations:** a LPNC, Univ. Grenoble Alpes, Univ. Savoie Mont Blanc, CNRS, 38000, Grenoble, France; b Institute for Transport Studies, University of Leeds, LS2 9JT, UK; c Université Clermont Auvergne, CNRS, LAPSCO, F-63000 Clermont-Ferrand, France; d Université Catholique de Louvain (UCLouvain), Psychological Sciences Research Institute, Louvain-la-Neuve, Belgium; e Fund for Scientific Research (FNRS-FRS), Brussels, Belgium

**Keywords:** Attentional blink, Visual consciousness, Spatial frequencies, Emotional facial expressions

## Abstract

In this article, we tested the respective importance of low spatial frequencies (LSF) and high spatial frequencies (HSF) for conscious visual recognition of emotional stimuli by using an attentional blink paradigm. Thirty-eight participants were asked to identify and report two targets (happy faces) embedded in a rapid serial visual presentation of distractors (angry faces). During attentional blink, conscious perception of the second target (T2) is usually altered when the lag between the two targets is short (200–500 ms) but is restored at longer lags. The distractors between T1 and T2 were either non-filtered (broad spatial frequencies, BSF), low-pass filtered (LSF), or high-pass filtered (HSF). Assuming that prediction abilities could be at the root of conscious visual recognition, we expected that LSF distractors could result in a greater disturbance of T2 reporting than HSF distractors. Results showed that both LSF and HSF play a role in the emergence of exogenous consciousness in the visual system. Furthermore, HSF distractors strongly affected T1 and T2 reporting irrespective of the lag between targets, suggesting their role for facial emotion processing. We discuss these results with regards to other models of visual recognition. .

## 1 Introduction

**Figure 1:**
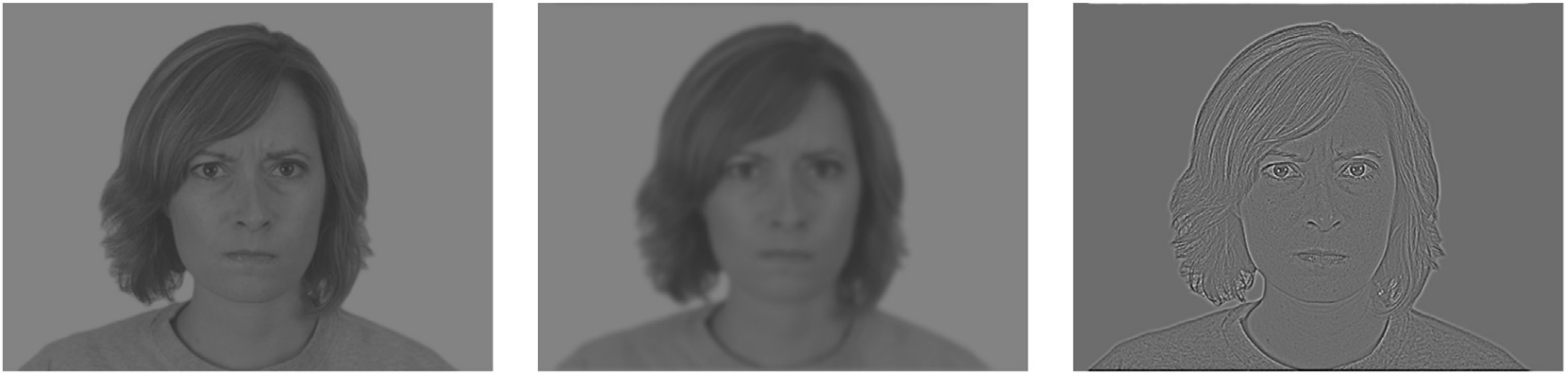
Examples of distractor stimuli. From left to right: broad spatial frequency (BSF), low spatial frequency (LSF), and high spatial frequency (HSF) version of an angry face.

Current models of cognition emphasize the role of predictions as a universal principle of the brain governing aspects such as visual processing and recognition (e.g. Bar, 2009a, 2009b; Friston and Kiebel, 2009; Kok and de Lange, 2015; Summerfield and Egner, 2009). The brain would routinely generate predictions about the world based on sensorial inputs and prior knowledge. More than mere identification, visual perception should be considered as a constant recognition process. Thus, instead of asking ‘what is this I am seeing?‘, the question could be ‘from what I already know, what does this look like?’ (Bar, 2009b). In the context of vision, it was suggested that information of high temporal resolution but low spatial frequencies (LSFs; fast coarse visual information; see Figure 1) would rapidly reach the orbitofrontal cortex (OFC) where hypotheses about the nature of the visual input would be generated. This would trigger top-down modulations towards occipito-temporal areas to guide the parallel processing of low temporal resolution and high spatial frequencies information (HSFs; slower fine visual information) (Bar et al., 2006; Chaumon et al., 2014; Kauffmann et al., 2015a, Kauffmann et al., 2015b, Kauffmann et al., 2015c; Kauffmann et al., 2015a, Kauffmann et al., 2015b, Kauffmann et al., 2015c; Kveraga et al., 2007; Peyrin et al., 2010). This anticipatory process could be akin to attentional mechanisms as it biases subsequent processing of the visual input, possibly through the inhibition of alternative interpretations (Bar, 2009b), and has been hypothesized as driving the emergence of visual consciousness (Bar et al., 2006; Breitmeyer, 2014; Kveraga et al., 2007). Importantly, these empirical data supporting the predictive brain hypothesis are intimately linked to the conscious recognition of the stimuli. Therefore, we assume that this top-down activity, based on LSF information, from the OFC to the occipito-temporal cortex could be at the root of conscious visual recognition of exogenous stimuli. However, whereas previous studies used simple visual recognition tasks to address this question (i.e. the participants have to explicitly report their conscious perception of the stimulus, as in Bar et al., 2006), we used a more implicit paradigm of attentional blink (Aguado et al., 2010). The advantage of the AB described below is that it allows investigating the emergence of conscious visual recognition by means of an implicit task that is very difficult to control consciously since it induces a transient blindness of the target stimulus to consciousness. This blindness of conscious perception is irrepressible and thus avoids explicit biases linked to a simple recognition task. Moreover, the AB allows exploring the temporal dynamic of this access to conscious recognition since this effect is transient (from 200 to 500 m, approximately). The aim of this paper is therefore to contribute to determining to what extent LSFs and HSFs are determinant for conscious recognition of visual stimuli.

To tackle this question, we used an attentional blink paradigm derived from a rapid serial visual presentation (RSVP) in which participants are presented with a number of images streaming sequentially at a frequency of about 100 m/image. The task is usually to detect and report two target images (T1 and T2) per trial, which are embedded between distractor images. Depending on the lag between T1 and T2 -that is, the number of distractors between them- T2 can be processed without ever reaching conscious report given T1 was indeed perceived. This phenomenon, called ‘attentional blink’ (Raymond et al., 1992), has been shown to occur using lags of approximately 200–500 ms between T1 and T2 (Luck et al., 1996; Martens and Valchev, 2009). This paradigm was thus relevant as it enabled us to manipulate the conscious report of visual stimuli.

The boost and bounce theory (Olivers and Meeter, 2008) states that a template of the targets—defining ‘the representations involved in the selection of task-relevant stimuli’ (Corbetta and Shulman, 2002, p. 202)—is used to modulate the bottom-up processing of visual input through top-down re-entrant connections according to the relevance of the visual information. Once a target is captured by the visual system, excitatory feedback is triggered from the frontal lobes back to the sensory areas in which the target is processed, enhancing its identification. Due to the latency of this activity, the distractors following T1 benefit from this boost, and the bounce (i.e. inhibitory feedback) produced by the distractors due to their irrelevancy is consequently boosted. When T2 finally appears, strong inhibitory feedback reaches the visual areas, resulting in an insufficient activation of T2. Obviously, distractors are critical for the occurrence of this phenomenon (see Olivers and Meeter, 2008) and the more the distractors share similarities with T1 (Müsch et al., 2012), the stronger the blink of T2. In other words, the key components of the attentional blink are the distractors that constitute a type of masking effect disrupting the processing of T2. This process implies that the higher the visibility of the distractors (modulated at different spatial frequencies), the higher the attentional blink, and the lower the performance on T2.

In order to investigate the role of LSF and HSF in the emergence of conscious perception of a visual stimulus within the framework of attentional blink paradigm, we presented inter-target distractors using different spatial frequency filters. We expected a stronger blink (i.e. a lower performance in detecting T2) for LSF rather than HSF distractors. LSFs would rapidly allow a conscious recognition of the inter-target distractors, which would in turn engender a strong bounce of the visual input through top-down modulations and interfere with T2's conscious report (since LSF distractors are not congruent with T1 and T2 emotional expressions). Conversely, information from HSF distractors would result in a reduced effect on the further processing of T2. The strongest blink was expected when the distractors were in broad spatial frequency (BSF), given they were intact and contained more information than the two other conditions. Finally, we expected an attentional blink and an interaction toward a reduction of this effect of the LSF distractors that should be reduced as the lag increased.

## 2 Material and methods

### 2.1 Participants

Thirty-eight undergraduates from the University of Grenoble Alpes (36 women, mean age = 20.97 years, SD = 5.94; 2 men, mean age = 22.5 years, SD = 2.12), left- or right-handed, participated in our experiment for course credits. All had normal or corrected-to-normal vision. There are similar studies conducted on as little as 12 participants (Nieuwenhuis et al., 2008) or at least as much as 55 participants (Vermeulen, 2010). We ran a power analysis based on ANOVA analyses with a small effect (Cohen's d = .20) with α = .05 and power = .95. The estimated N was 27. This sample size is in line with a recent replication study of the attentional blink using ANOVA and bayesian mixed model analyses (Grassi et al., 2020). However, we were not able to run a power analysis regarding the spatial frequency effect due to the novelty of the paradigm. This effect is expected to be more subtle, inviting us to include more than 27 participants.

### 2.2 Apparatus

The experiment was performed using the E-Prime 2 software (E-Prime Psychology Software Tools Inc., Pittsburg, PA) on an HP Z400 Workstation computer, Intel Xeon CPU, running Microsoft Windows XP, and plugged into a Dell M783p 17″ CRT monitor (16” diagonal viewable size) set at a resolution of 1024 × 768 pixels (aspect ratio 4:3) with a refresh rate of 100 Hz via VGA port. Participants were tested at a viewing distance of 100 cm with their head placed on a chinrest and wearing a noise cancelling Pelter Optime III headset.

### 2.3 Stimuli

We used emotional facial expressions (EFEs) stimuli in order to strengthen the potential implication of the OFC, which has also been shown to be strongly involved in processing emotional stimuli (Barrett and Bar, 2009; Beffara et al., 2015). Stimuli were high-resolution 256-level grey-scale photographs from the Chicago Face Database (Ma et al., 2015) cropped from a 2444 × 1718 pixel size to a 2288 × 1716 pixel size (4:3 aspect ratio, 18.4 ° × 13.8 °). Images were also converted from a JPEG format into a BMP format in order to standardize file size and loading time in the E-Prime software. Forty faces (20 females and 20 males) expressing either happiness or anger were used as targets and distractors, respectively. Angry faces could be presented in three forms: BSF, LSF, and HSF. The spatial frequency content was filtered by multiplying the Fourier transform of the original images by the kernel of LSF and HSF filters. For LSF stimuli, spatial frequencies above 2 cycles per degree (cpd; i.e. low-pass cut-offs of 36.89 cycles per image width) were removed, corresponding to 10.48 cycles per face (cpf). For HSF stimuli, spatial frequencies below 6 cpd were removed (i.e. high-pass cut-offs of 110.69 cycles per image), corresponding to 31.44 cpf. To avoid any influence by the following features on the spatial frequency processing, the mean luminance was equalized for each filtered stimulus by attributing the same mean luminance (0.5 on a scale from 0 to 1), and the root mean squared (RMS) contrast was set to 0.1 (Kauffmann et al., 2015; Vlamings et al., 2009).

### 2.4 Procedure

**Figure 2:**
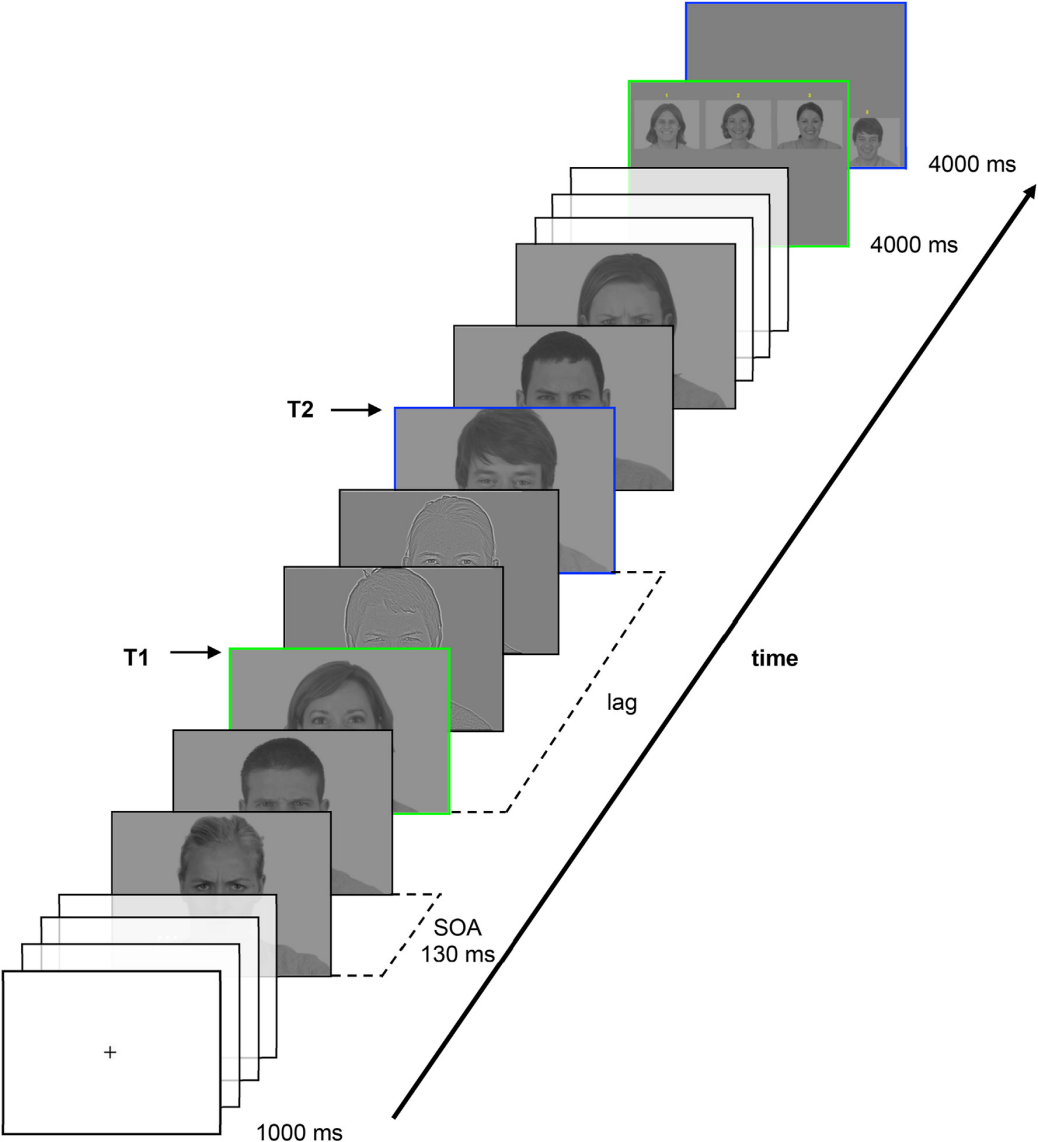
Example of a trial with high-pass filtered (HSF) stimuli as inter-target distractors. Green/Light grey edges indicate the first target (T1) and the slide where it was to be retrieved. Blue/Dark grey edges indicate the slides for the second target (T2). The lag corresponds to the onset of T2 (here, ‘Lag 3’) relative to T1's onset (also called ‘Lag 0’).SOA = stimulus onset asynchrony.

Participants were tested individually in a darkened experimental box. They were instructed to detect two happy faces (targets) among angry faces (distractors) all presented sequentially at a quick pace. Because the attentional blink is inherently a difficult paradigm that may result in floor effects, we chose to use happy faces as targets, which have been shown to be easier to detect than other emotional expressions (Calvo and Nummenmaa, 2016; Mermillod et al., 2011). To ensure that participants understood the instructions, the experiment started with a short training phase of 8 trials split into two conditions that were absent from the experimental session: a ‘no-T2-with-BSF-distractors’ condition (simulating an attentional blink) and a ‘lag-9-with-BSF-distractors’ condition where T2 was out of the usual blink period. Each trial started with a fixation cross lasting 1 s followed by a 13- to 20-image-long rapid serial visual presentation (RSVP) at a frequency of about ≈7.69 Hz (each image lasting 130 m). T1 was presented either after four, five, six, or seven non-filtered distractors, and two, four, or six distractors were embedded between T1 and T2. Therefore, the onsets of T2 corresponded to lags 3, 5, and 7 respectively, or onset times of 390 m, 650 m, and 910 m (lag 0 being the onset of T1). The experiment included 180 trials: 20 trials for each T2 lag (lag 3, lag 5, and lag 7) and each spatial frequency content of distractors (BFS, LSF, and HSF). For each trial, all inter-target distractors were similarly filtered and the number of distractors left after T2 was fixed at five in order to reduce the variance in content degradation in short-term memory before target retrieval (see Figure 2). For each trial, faces stimuli were drawn randomly from our 40-face database: four to six faces as potential non-filtered distractors before T1, one face as T1, four to six faces as the potential inter-target distractors (BFS, LSF, or HSF), one face as T2, and always five faces as distractors at the end. The same trials were used for all participants, but trials were randomized between participants using E-Prime software in order to avoid a familiarity effect of a face. For each trial, all inter-target distractors were similarly filtered and the number of distractors left after T2 was fixed at five in order to reduce the variance in content degradation in short-term memory before target retrieval (see Figure 2).

At the end of each trial, participants were first prompted to identify T1, presented along with two other possible targets, by pressing keys ‘1’, ‘2’, or ‘3’ on a numeric pad with the three middle fingers of their right hand. These three potential targets were displayed horizontally at the top of the screen with a digit above each to indicate the corresponding key to press. Participants were given 4 s to provide their answer. A similar screen was displayed for the same maximal duration for the retrieval of T2. However, the targets were presented at the bottom of the screen to ensure that participants noticed the screen had changed in case no response had been recorded for the retrieval of T1 or in case the screen change had occurred during an eye saccade or head movement (i.e. saccadic suppression). In case no target was perceived or recognized, participants were instructed to press the spacebar with their left hand rather than answering randomly. In this case, the answer was considered as a not consciously perceived response.

The study lasted around 20 min, was introduced as investigating consciousness and attention, and corresponded to a 3 (filter of inter-targets distractors: BSF, LSF, and HSF) × 3 (T2 lag: 3, 5, and 7) fully within-subject design.

### 2.5 Data analysis

Statistical analyses were conducted using the *lme4* package (Bates et al., 2015) implemented in the R software (R Development Core Team, 2016) to run a generalized linear mixed effects logistic regression on the accuracy of T2 report given that T1 was correctly reported (T2|T1). These statistical analyses are used to model binary outcome variables (e.g. where 0 is failure and 1 is success), which are not characterized by a normal distribution but rather by a binomial distribution. The logistic regression is therefore designed to transform the nonlinear relationship of our predictors with our dependent variable into a linear relation as described in Eq. (1).

(1) $$logit\left(p\right)=\ln\left(\frac{p}{1-p}\right)=\beta_{0}+\beta_{1}x_{1}+\ldots +\beta_{n}x_{n}$$

where *p* is the probability of our dependent variable being equal to 1, that is, the probability of success.

Categorical variables are generally included in logistic regression using dummy variables which have the advantage to lead to interpretations of the exponential of the parameters (*e*^*β*^) in terms of odds ratios (e.g. Jaccard, 2001). For example, if in a given condition, T2 was reported successfully 50 out of 100 times while in the reference condition T2 was reported correctly 25 out of 100 times, the odds of T2 being correctly reported is 50/50 = 1 in the first condition and 25/75 = .33 in the reference condition. The odds ratio between these two conditions, 1/0.33 = 3, indicates that the odds of T2 being reported are 3 times higher in the first condition than in the reference condition. It is given by the exponential of the parameter (*e*^*β*^) of the dummy variable such as 1 indicates the condition to be compared and 0 the other conditions (the reference condition being coded 0 for all dummy variables). As conditions are only compared one by one to the reference condition in the dummy coding scheme, this is insufficient when more complex comparisons are necessary (e.g. one group vs. two other groups). In those cases, it is possible to use contrasts variables in place of dummy variables. However, the exponential of the parameters (*e*^*β*^) of the contrast variables are linked in a more complex way to odds ratios (e.g. Hosmer et al., 2013) and the parameters *β* themselves are better suited to interpret for comparisons. They indeed have the same interpretations as they have in linear models but expressed for the transformed variable logit. For example, if the contrast (1, −1/2, −1/2) is used to compare condition 1 to conditions 2 and 3, the interpretation of the corresponding β will be the logit in condition 1 minus the mean of the logit of undifferentiated conditions 2 and 3. As the function logit is an increasing function of accuracy (*p*_*i*_), this also allows to test for accuracy differences.

The residuals from a linear regression with the same contrasts used for our logistic regression were tested for normality of distribution using the Shapiro-Wilk test; it resulted that the distribution was significantly different from that of a normal distribution, W = 0.989, *p* = .012. After removal of four participants, whose accuracy never exceeded 15% in any of the three lag conditions, the distribution was not significantly abnormal, W = 0.995, *p* = .39.

#### 2.5.1 Fixed effects

As fixed effects, we entered two orthogonal Helmert contrasts for the effect of the lags, two orthogonal Helmert contrasts for the effect of the spatial frequency of distractors, and the four interaction terms (see Table 1).

**Table 1:** Contrasts used in our statistical analyses. Ψ stands for contrast. “Dist.” is short for “Distractor”.

	Lag 3	Lag 5	Lag 7	Lag 3	Lag 5	Lag 7	Lag 3	Lag 5	Lag 7
	BSF			LSF			HSF		
Lag *Ψ*_1_	−0.5	0.25	0.25	−0.5	0.25	0.25	−0.5	0.25	0.25
Lag *Ψ*_2_	0	−0.5	0.5	0	−0.5	0.5	0	−0.5	0.5
Dist. *Ψ*_1_	−0.5	−0.5	−0.5	0.25	0.25	0.25	0.25	0.25	0.25
Dist. *Ψ*_2_	0	0	0	−0.5	−0.5	−0.5	0.5	0.5	0.5
Lag *Ψ*_1_ × Dist. *Ψ*_1_	0.25	−0.125	−0.125	−0.125	0.0625	0.0625	−0.125	0.0625	0.0625
Lag *Ψ*_1_ × Dist. *Ψ*_2_	0	0	0	0.25	−0.125	−0.125	−0.25	0.125	0.125
Lag *Ψ*_2_ × Dist. *Ψ*_1_	0	0.25	−0.25	0	−0.125	0.125	0	−0.125	0.125
Lag *Ψ*_2_ × Dist. *Ψ*_2_	0	0	0	0	0.25	−0.25	0	−0.25	0.25

The *lmerTest* package was used to obtain *p*-values associated with the fixed effects (Kuznetsova et al., 2017). For more information about mixed models see Judd et al., 2012, Judd et al., 2017. The significance threshold was set at α = .05.

#### 2.5.2 Random effects

By-subject intercepts, by-T2 intercepts, and by-T1 intercepts were estimated as random effects. Random slopes and covariances were not estimated.

The resulting model is described in Eq. (2).

(2) $$Accuracy=Lag\;\Psi_{{}_{1}}+Lag\;\Psi_{2}+Dis\;\Psi_{1}+Dis\;\Psi_{2}+\left(interactions\right)+\left(Participant\right)+\left(T2\right)+\left(T1\right)$$

## 3 Results

**Figure 3:**
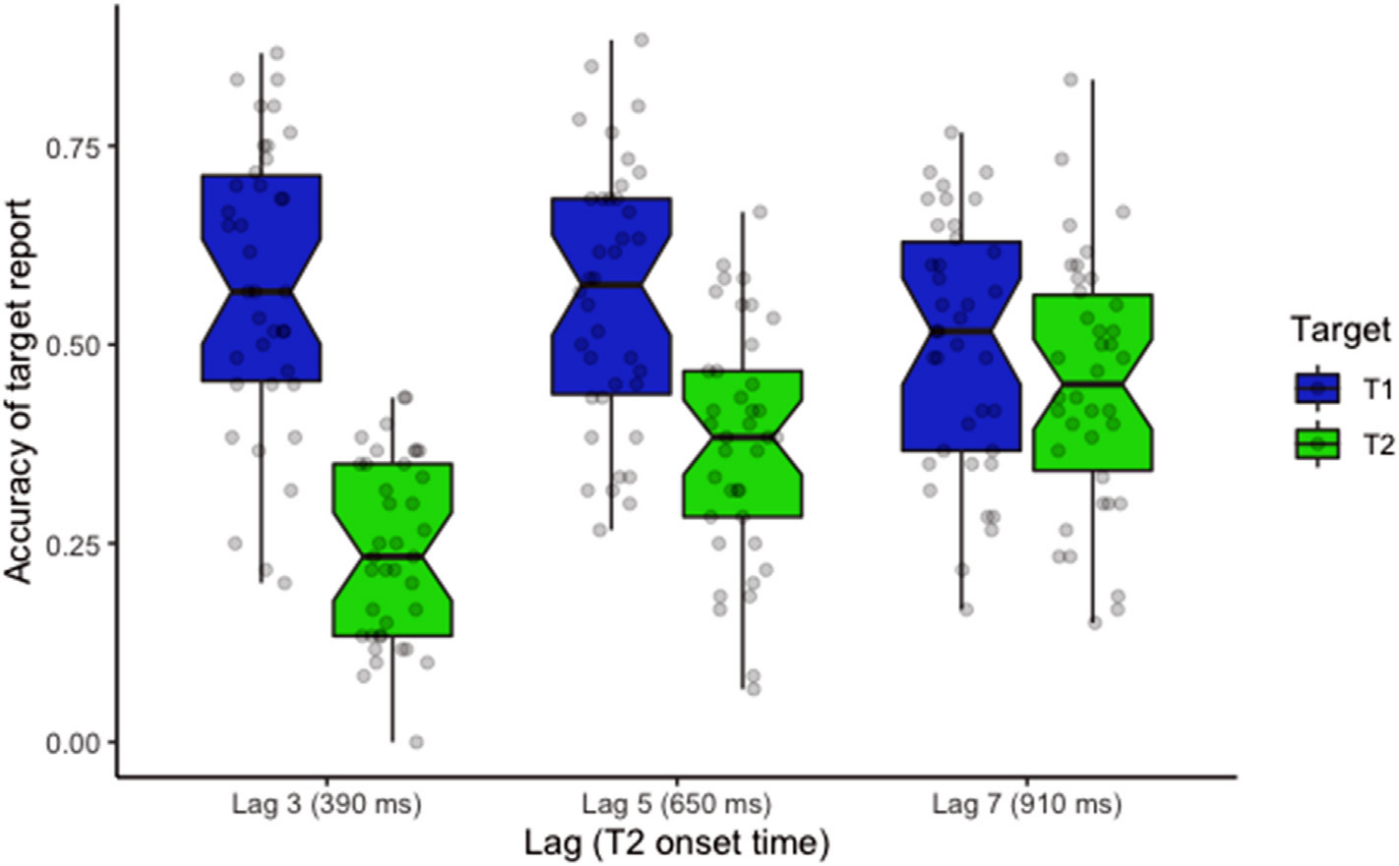
Boxplot represents the accuracy for T1 and T2 reports in lags 3 to 7 conditions.

Accuracy (percentage of correct responses) of T1 and T2 report in the different lag conditions is shown on Figure 3. The attentional blink phenomenon clearly appears as the difference between both targets’ report decreases the longer the lag.

**Figure 4:**
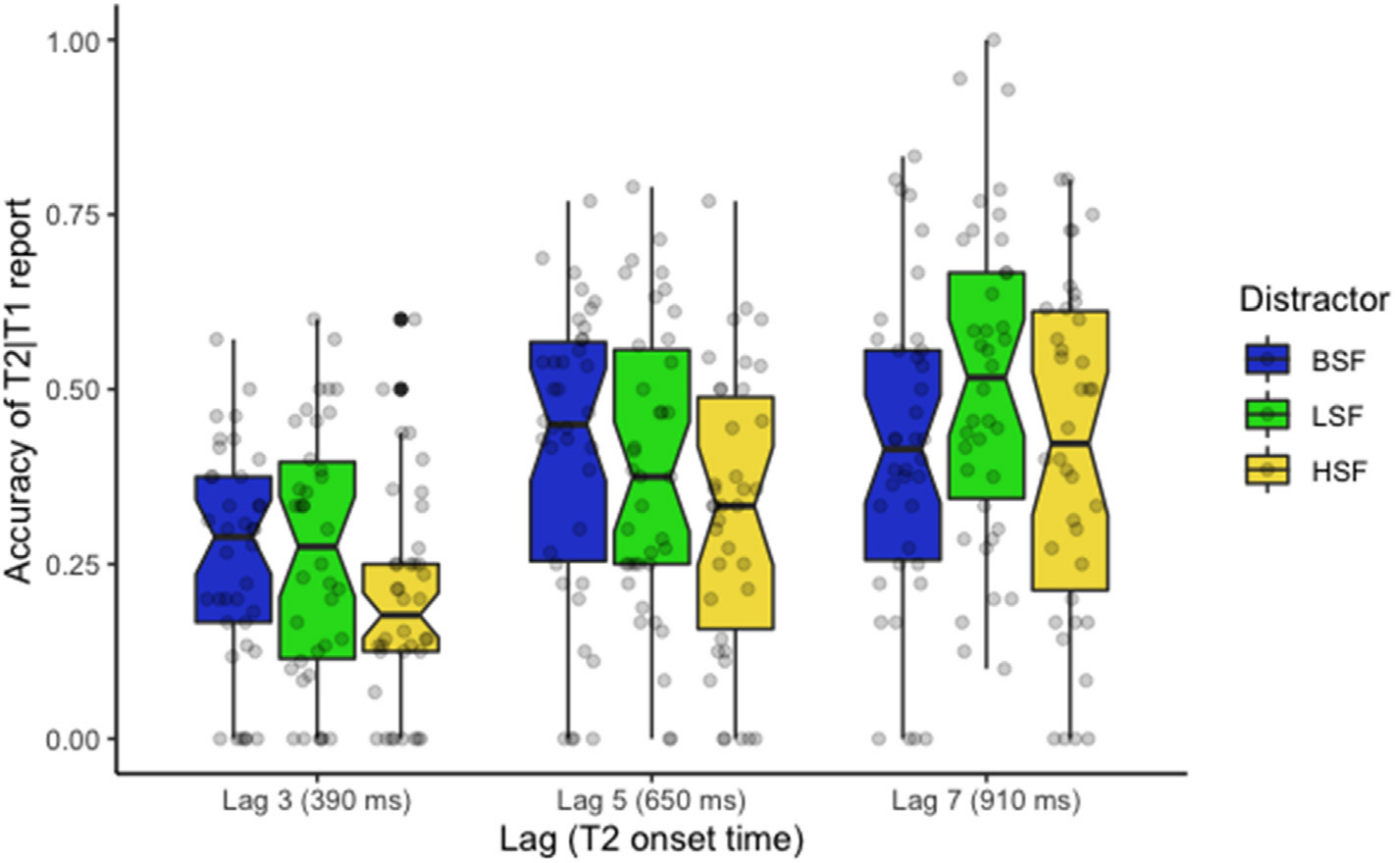
Boxplot represents the accuracy of T2|T1 report for lags 3, 5, and 7 when the inter-target distractors were broad-spatial frequency (BSF), low-spatial frequency (LSF), and high-spatial frequency (HSF) faces.

Accuracy of T2 report given T1's correct report (T2|T1) are reported in Table 2 and shown on Figure 4. The results of our logistic regression are reported in Table 3. The first contrast for the lags' effect (Lag Ψ1) was significant (*β* = 1.32, 95% CI 1.06 to 1.58, *p* < .001) indicating that the accuracy of T2 being correctly reported is higher at lags 5 or 7 than at lag 3. The second contrast (Lag *Ψ*_2_) was also significant (*β* = .23, 95% CI .01 to .45, *p* = .039), indicating that T2 was less likely to be correctly reported at lag 5 than 7. These results demonstrate the presence of an attentional blink as the performance in detecting T2 increased with increasing lag. Regarding the distractors, our first comparison (Dist. *Ψ*_1_) revealed a non-significant difference between BSF and the filtered distractors, whereas our second comparison (Dist. *Ψ*_2_) revealed a significant difference between LSF and HSF independent of the lags (*β* = −.42, 95% CI −.63 to −.20, *p* < .001), indicating a better report of T2 when the inter-target distractors were low-pass filtered (LSF) than when they were high-pass filtered (HSF). The interaction between the second contrast of the lag and the second contrast of distractors (Lag *Ψ*_2_ × Dist. *Ψ*_2_) was also significant (*β* = −.52, 95% CI −1.02 to −.03, *p* = .037), indicating that a disruptive effect of the HSF on visual recognition was observed beyond the usual time window of the AB. The interaction between the second contrast of the lag and the first contrast of distractors (Lag *Ψ*_2_ × Dist. *Ψ*_1_) was marginally significant (*β* = .55, 95% CI −0.09 to 1.19, *p* = .092). It suggests that filtered distractors produced a smaller blink at lag 7 than at lag 5 compared to non-filtered distractors (i.e. LSF and HSF vs. BSF).

**Table 2:** Percentage (and standard error) of correct T2|T1 report per condition.

Distractors	Lag 3	Lag 5	Lag 7
BSF	26.8% (2.50)	45.0% (3.20)	45.7% (3.35)
LSF	29.7% (2.83)	41.8% (3.21)	55.0% (3.61)
HSF	21.4% (2.56)	34.6% (3.29)	45.0% (3.83)
Mean	26.0% (2.07)	41.1% (2.35)	49.3% (2.71)

**Table 3:** Fixed effects of the logistic regression analysis of T2 |T1 reports. Asterisks ‘∗’ indicate significant results. Middle dots ‘·’ indicate trends.

	β	*SE* β	CI 95%	Wald's *z*	*p*	
(Intercept)	−0.73	0.17	−1.05 – −0.40	−4.39	<.001	∗
Lag *Ψ*_1_	1.32	0.13	1.06–1.58	9.94	<.001	∗
Lag *Ψ*_2_	0.23	0.11	0.01–0.45	2.07	.039	∗
Dist. *Ψ*_1_	0.00	0.13	−0.26–0.26	−0.01	0.994	
Dist. *Ψ*_2_	−0.42	0.11	−0.63 – −0.20	−3.84	<.001	∗
Lag *Ψ*_1_ × Dist. *Ψ*_1_	0.39	0.36	−0.31–1.10	1.09	0.275	
Lag *Ψ*_1_ × Dist. *Ψ*_2_	0.14	0.31	−0.47–0.74	0.44	0.663	
Lag *Ψ*_2_ × Dist. *Ψ*_1_	0.55	0.33	−0.09–1.19	1.69	0.092	**·**
Lag *Ψ*_2_ × Dist. *Ψ*_*2*_	−0.52	0.25	−1.02 – −.03	−2.08	0.037	∗

**Figure 5:**
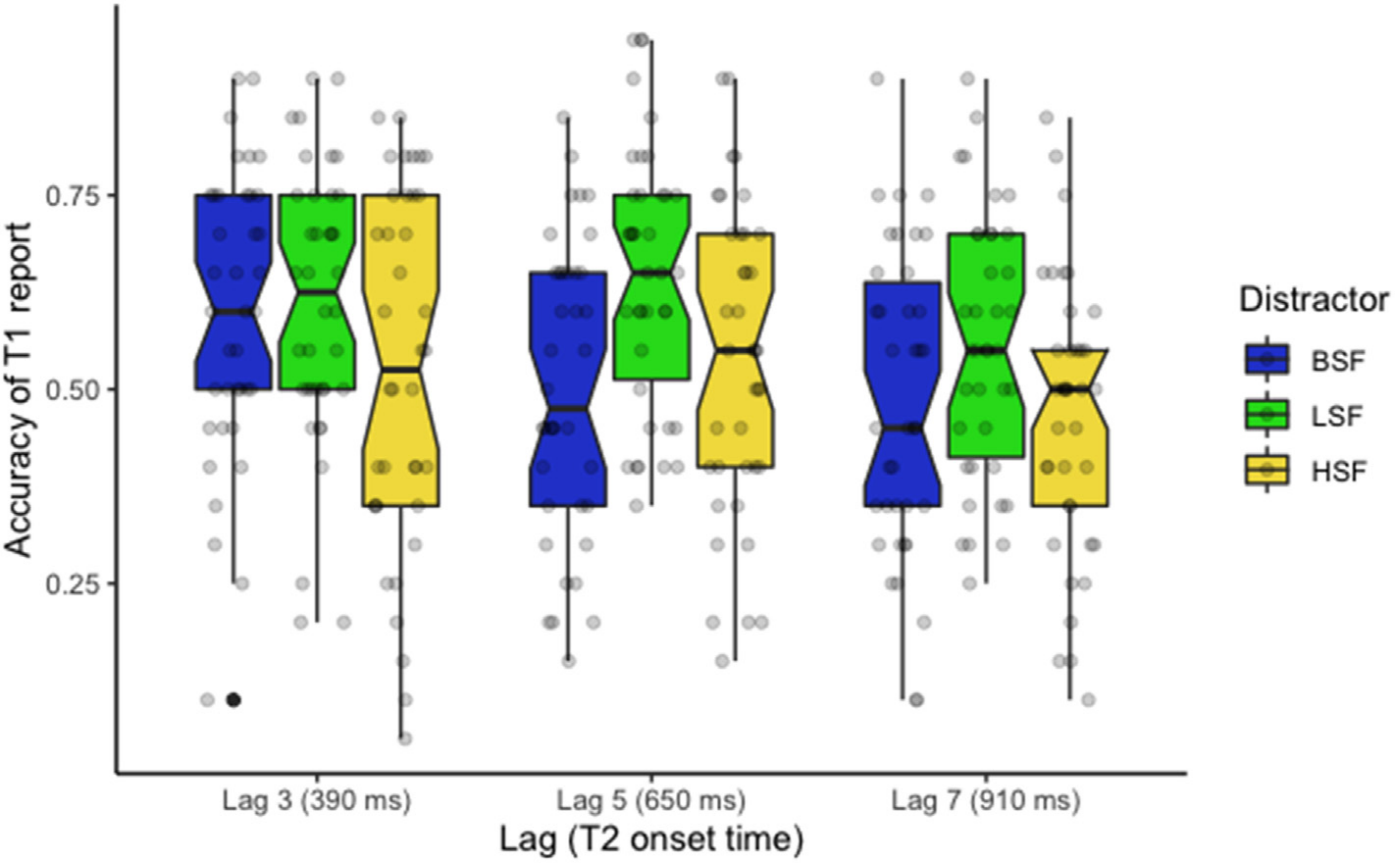
Boxplot represents the accuracy of T1 report for lags 3, 5, and 7 when the inter-target distractors were broad-spatial frequency (BSF), low-spatial frequency (LSF), and high-spatial frequency (HSF) faces.

Complementary to our princeps analyses related to our a priori analysis, we ran an exploratory analysis on the report of T1. Accuracy of T1 is shown on Figure 5. The results of our logistic regression are reported in Table 4. The first contrast for the lags’ effect (Lag Ψ1) was significant (*β* = −.38, 95% CI −.56 to −.20, *p* < .001), indicating that T1 was more likely to be correctly reported at lag 3 than at lags 5 or 7. The second contrast (Lag Ψ2) was also significant (*β* = −.32, 95% CI −.48 to −.16, *p* < .001), indicating that T1 was more likely to be correctly reported at lag 5 than 7. Regarding the distractors, our first comparison (Dist. Ψ1) revealed a non-significant difference between BSF and the filtered distractors, whereas our second comparison (Dist. Ψ2) revealed a significant difference between LSF and HSF independent of the lags (*β* = −.40, 95% CI −.56 to −.24, *p* < .001), indicating a better report of T1 when the inter-target distractors were low-pass filtered (LSF) than when they were high-pass filtered (HSF). The interaction between the first contrast of the lag and the first contrast of distractors (Lag Ψ1 × Dist. Ψ1) was significant (*β* = .67, 95% CI .18 to 1.17, *p* = .008), suggesting that the difference of T1 report between filtered distractors and non-filtered distractors (i.e. LSF and HSF vs. BSF) was larger at lag 5 or 7 compared to lag 3. The interaction between the second contrast of the lag and the first contrast of distractors (Lag Ψ2 × Dist. Ψ1) was also significant (*β* = −.49, 95% CI −.94 to −.03, *p* = .036) suggesting that the difference between filtered distractors and non-filtered distractors (i.e. LSF and HSF vs. BSF) was lower at lag 7 compared to lag 5.

**Table 4:** Fixed effects of the logistic regression analysis of T1 reports. Asterisks ‘∗’ indicate significant results.

	β	*SE* β	CI 95%	Wald's *z*	*p*	
(Intercept)	0.20	0.16	−0.12–0.52	1.22	0.224	
Lag *Ψ*_1_	−0.38	0.09	−0.56 –−0.20	−4.11	<.001	∗
Lag *Ψ*_2_	−0.32	0.08	−0.48 – −0.16	−3.88	<.001	∗
Dist. *Ψ*_1_	0.05	0.09	−0.13–0.24	0.54	0.590	
Dist. *Ψ*_2_	−0.40	0.08	−0.56 – −0.24	−5.02	<.001	∗
Lag *Ψ*_1_ × Dist. *Ψ*_1_	0.67	0.25	0.18–1.17	2.65	0.008	∗
Lag *Ψ*_1_ × Dist. *Ψ*_2_	0.00	0.21	−0.42–0.42	0.00	0.996	
Lag *Ψ*_2_ × Dist. *Ψ*_1_	−0.49	0.23	−0.94 – −0.03	−2.10	0.036	∗
Lag *Ψ*_2_ × Dist. *Ψ*_*2*_	0.16	0.19	−0.22–0.53	0.82	0.409	

## 4 Discussion

Our study was designed to investigate the importance of spatial frequencies in the emergence of visual perception via a modulation of the attentional blink effect. Since LSFs are assumed to be critical for triggering top-down modulations of the visual information processing (Bar, 2009b; Breitmeyer, 2014), we hypothesized that the LSF content of inter-target distractors during an RSVP would increase the attentional blink through a stronger interference induced by LSF distractors (compared to HSF distractors) on the conscious perception of target stimuli.

We did observe an attentional blink, as evidenced by the lower probability of reporting T2 the shorter the lag independently of the filter applied to the distractors. However, contrary to our predictions, participants more often failed to report T2 when the inter-target distractors were HSF than when they were LSF.

These results are in sharp contrast with previous theoretical frameworks suggesting that top-down signals initiated by LSF information processing are the basis of the conscious perception of visual stimuli (Bar, 2009b; Breitmeyer, 2014). Conscious perception of the target was more disrupted by HSF distractors, frequencies that are predominantly processed within the ventral stream. These results contradict our initial hypothesis assuming that exogenous consciousness was based on a top-down model of visual perception (Bar, 2003) on the basis of LSF information conveyed rapidly from the OFC to the ventral stream (i.e. the occipito-temporal pathway). However, results clearly indicate that LSF, and more importantly HSF information, processed exclusively by the ventral stream, induced an attentional blink and therefore that both ranges of spatial frequencies are important for exogenous consciousness albeit the fact that HSF information deteriorates more heavily the conscious perception of T2 and T1 independently of the lag. It should be noted that the ventral stream, unlike the dorsal stream, is a more balanced combination of magno-, konio-, and parvocellular pathways (Merigan et al., 1991), but also that HSF is uniquely processed by this neural pathway, potentially explaining the difference between the BSF and LSF conditions at lags 3 and 5.

This unexpected effect of HSF raises important questions for further studies. A first basic assumption is that the effect of HSF on the AB could be related to a basic perceptual effect, for instance because HSF faces could constitute more unusual and harder-to-identify stimuli than LSF faces, resulting in stronger AB. However, a simple perceptual artifact is unlikely given that we applied a filtering procedure similar to almost all scientific papers in this field of research: we used the exact same gaussian filters as any other experimental studies investigating the effect of SF processing and we removed the lowest SF channel in order to have more similar stimuli for each SF channels. This precaution is rarely applied in other studies. Indeed, without this precaution, LSF stimuli are made of big black and white blobs that are very different at a perceptual level compared to HSF stimuli. Moreover, the human retina is naturally exposed to the integral SF range. Therefore, it is difficult to support the claim that HSF were more surprising or harder to identify than LSF faces. Alternatively, this effect of the HSFs on the AB could be related to a higher capture of attention by HSF information, potentially related to a higher pro- or retroactive masking effect of the targets (on T2 or T1) or, in a more fundamental perspective, an intimate link between the ventral stream processing exclusively HSF information and conscious perception. Further studies will have to address these different hypotheses in order to determine if this effect is related to basic perceptual factors (e.g. higher masking effect of HSFs) or a more intrinsic link between HSF pathways of the neural underpinnings of attention and/or visual consciousness.

Additionally, the current data suggest that visual consciousness is not necessarily founded in this LSF-based top-down activity from the OFC to the infero-temporal (IT) cortex, which may only allow implicit processes based on predictive capacities but not conscious visual perception per se. In other words, we do not claim definite assumptions about the bottom-up (versus top-down) nature of this effect. Evidence obtained using electrophysiological methods suggests that conscious perception of exogenous stimuli could be achieved within the temporal pathway (Navajas et al., 2013; Quiroga et al., 2008), and showed the importance of HSF information for the conscious recognition of visual stimuli (Tian et al., 2018; You and Li, 2015). Yet, even if our current data appears to be in accordance with these findings, we hereby do not provide indisputable evidence supporting this link between HSF neural pathways and exogenous visual consciousness. Moreover, while we found a main effect of the Lag suggesting that both LSFs and HSFs play a role in the attentional blink, results indicated an absence of interaction effect between spatial frequencies and lags, showing that the disruption of T2 by HSF stimuli continues after the lags involved in the attentional blink. This result suggests that this effect is not specific to the attentional blink (since the interaction is not significant) and rather indicates that HSFs influence the subsequent processing of a non-filtered visual stimuli irrespective to the lags characterizing the attentional blink. This main effect of both LSF and HSF filtering is not absolutely surprising since Nieuwenstein et al. (2009) have shown that the attentional blink could also occur, albeit weaker, without distractors, when presenting a blank screen between T1 and T2. In other words, our interest is more focused on the higher disturbance from the HSF distractors than on the absolute AB effect of both spatial frequency ranges. This effect is consistent with findings from previous studies using different stimuli or tasks, suggesting a prominent role of HSFs during facial emotion processing (Lacroix et al., 2021; Dutheil, 2021; Perrier, 2021; Shankland et al., 2021). However, as suggested above, it cannot be ruled out that impaired detection of T2 following HSF distractors regardless of the lag simply reflects an effect of forward masking by HSF stimuli or a potential familiarity effect (Endress and Potter, 2014). A potential explanation for this is that the contrast of HSF and LSF distractors was equalized to an RMS of 0.1, resulting in HSF distractors having a higher contrast than unfiltered stimuli (HSFs usually have a RMS contrast around 0.05). Thus, the higher contrast of HSF information in distractors than in the Targets may have resulted in HSF distractors masking the HSF content of T2. Critically, in the present experiment, this masking effect could have been detrimental to the processing of T2 non-filtered faces, suggesting that the HSF content is the spatial frequency range preferentially used to resolve our task (namely, the conscious recognition of a stimulus). Indeed, previous behavioral experiments (Schyns and Oliva, 1999), as well as data from computational neurosciences (Mermillod et al., 2010; Mermillod et al., 2005), have shown that different spatial frequencies could be more or less useful (i.e. diagnostic) for different tasks (either categorization or identification tasks). In our experiment, it seems that the HSF content was highly decisive for the conscious identification of the faces (e.g. Aguado et al., 2010; de Gardelle and Kouider, 2010; Gao and Maurer, 2011), hence, produced a greater disturbance on T2 report than the LSF content did. In this context, it is also possible that HSFs could be more diagnostic for the perception of angry distractors. For instance, the HSF signal around the eyes could be more useful to detect the wrinkles around the eyes characteristic of angry expressions (Smith et al., 2005). The better recognition of angry expressions in distractors could then impair the conscious recognition of individuals with happy expressions on T2 (and T1) at the end of the trials.

We ran a complementary analysis on T1 reports showing that HSF distractors are not only disturbing more heavily the perception of T2 during and after the blink, but also before the blink, affecting T1's accuracy. This surprising result could raise important questions with regards to competitive models of attentional blink. On the one hand, the current results confirm the importance of the distractors as suggested by Olivers and Meeter (2008) in the general framework of the boost and bounce theory since different distractors (either HSF or LSF) produced different disturbances in the explicit recognition of T1 and T2 (before, during, and after the attentional blink). According to this theory, the attentional blink is produced by the disruption of T2's perception caused by post-T1 distractors. Nonetheless, our results (especially at the level of T1 report) cannot completely eliminate T1-based theories suggesting that the attentional blink could be initiated by a T1 period of inattention (Nieuwenstein, Potter & Theeuwes, 2009; Lagroix et al., 2012) or by conscious perception of T1, a necessary but not sufficient condition to produce the attentional blink (Ophir, Hesselmann & Lamy, 2020). We may assume here that HSF distractors could decrease more strongly the conscious perception of T1 as well as T2 independently of the attentional blink.

One limitation of the experiment pertains to its design. Indeed, both T1 and T2 accuracy appeared reduced in our experiment compared to other attentional blink studies involving emotional faces (Eiserbeck & Abdel Rahman, 2020; Fox et al. 2005; Müsch et al., 2012), resulting in a reduced number of trials included in our analysis. Previous experiments sometimes used very dissimilar targets and distractors: for instance, Müsch et al. (2012) observed in four experiments a ceiling T1 accuracy as well as an attentional blink using upright faces as targets and inverted faces as distractors. However, using emotional faces as targets and unfiltered neutral upright faces as distractors in a fifth experiment they observed a low T1 accuracy but failed to observe an attentional blink (contrary to our experiment). In line with their studies, our results showed that high similarity between target and distractors could have impaired T1 and T2 report. It should also be noted that a recent study on the attentional blink conducted on 100 participants suggested that floor performances are not rare during this task (Grassi et al., 2020). Using inverted filtered distractors in future studies might help improving T1 accuracy while being also less ecological.

Finally, further neuroimaging studies combining high spatial and temporal resolution (e.g. iEEG) should be considered in order to disambiguate the possibility of top-down versus bottom-up nature of the effect, as top-down modulation of the attentional blink by the HSFs may also occur within the ventral stream or extra-striate cortical areas (Bullier, 2001).

## 5 Conclusion

This research is, to our knowledge, the first evidence of the importance of both LSFs and HSFs in the attentional blink, but stronger and widespread influence of HSFs, independent of the lags on conscious recognition. Previous works include the use of schematic faces (e.g. Maratos et al., 2008), double-task or double-template paradigms (e.g. Ricciardelli et al., 2012; see Soto-Faraco and Spence, 2002), scattered-faces as distractors (e.g. Bach et al., 2014), or the use of EFE as a means to modulate the blink of visually-basic stimuli (e.g. Vermeulen et al., 2009).

We would encourage further investigation on whether the spatial frequency content of both the targets and the distractors are determinant for the extent of the attentional blink effect. This investigation should include additional longer lags to determine when this effect fades. More specifically, the intriguing phenomenon showing that distractors modulate the attentional blink, but also that this effect persist for HSF distractors beyond the attentional blink is very important in order to understand the causal underpinnings of this effect (e.g. Goffaux et al., 2005).

## Declarations

### Author contribution statement

Martial Mermillod: Conceived and designed the experiments; Analyzed and interpreted the data; Wrote the paper.

Mickaël Jean Rémy Perrier: Conceived and designed the experiments; Performed the experiments; Analysed and interpreted the data; Wrote the paper.

Adeline Lacroix: Analysed and interpreted the data; Wrote the paper.

Louise Kauffmann; Carole Peyrin: Contributed reagents, materials, analysis tools or data; Analyzed and interpreted the data; Wrote the paper.

Alain Méot: Analyzed and interpreted the data; Wrote the paper.

Nicolas Vermeulen; Frédéric Dutheil: Conceived and designed the experiments; Analyzed and interpreted the data; Wrote the paper.

### Funding statement

This work has been partially supported by MIAI @ Grenoble Alpes, (ANR-19-P3IA-0003) to Martial Mermillod.

### Data availability statement

Data associated with this study has been deposited at https://osf.io/n4svb/.

### Declaration of interests statement

The authors declare no conflict of interest.

### Additional information

No additional information is available for this paper.
